# Primary squamous cell carcinoma, breast: A challenging diagnosis

**DOI:** 10.1002/cnr2.1391

**Published:** 2021-05-05

**Authors:** Divya Goel, Chanchal Rana, Suresh Babu, Pooja Ramakant

**Affiliations:** ^1^ Department of Pathology King George's Medical University Lucknow India; ^2^ Department of Endocrine surgery King George's Medical University Lucknow India

**Keywords:** aggressive, hormone, metaplastic, squamous cell carcinoma

## Abstract

**Background:**

Primary squamous cell carcinoma of the breast is an extremely rare malignancy constituting less than 0.1% of all breast cancers with very few cases reported in literature. It is an aggressive, triple‐hormone negative tumor, and its appropriate management is still debated. It is diagnostically challenging on both histopathology as well as radiology. Different diagnostic criteria are established for its definite diagnosis. As squamous cells are not found normally in the breast, various theories have been proposed regarding its origin. But the exact pathogenesis is still unclear. We report one such case encountered.

**Case:**

A 54‐year‐old female presented with gradually progressive painless lump in the right breast for 3 months with no other clinical features. There was neither any history of chronic or malignant disease in the patient nor in her family. On clinical examination, there was well‐defined, firm and nontender swelling in upper inner quadrant measuring 3 × 2 cm with overlying skin being normal. There was no swelling in the contralateral breast as well as in the bilateral axillary region. A suspicion of malignancy was raised on initial core needle biopsy and, on repeat biopsy, was diagnosed as metaplastic carcinoma with squamous differentiation. Later, on final resection, specimen was reported as primary squamous cell carcinoma of the breast without any nodal metastasis. All the metastatic causes were ruled out through proper clinical, radiological, and histopathological correlation.

**Conclusion:**

Primary squamous cell carcinoma of the breast is an aggressive tumor with its treatment protocol being still unclear, owing to its rarity. It is important to rule out the metastatic causes. It is relatively resistant to conventional chemotherapy, and its prognosis is also unpredictable. Hence, this requires further studies in terms of management and prognosis.

## 1 INTRODUCTION

The World Health Organization (WHO) defines primary squamous cell carcinoma (SCC) of the breast as a type of metaplastic carcinoma which is entirely composed of metaplastic squamous cell with variable keratin. Different diagnostic criteria are established for its definite diagnosis. It is diagnostically challenging on both histopathology as well as radiology. It is a very rare entity, constituting <0.1% of invasive breast cancer, with very few reported cases in literature so far.^1^ This tumor is described as an aggressive, hormone‐negative, and treatment refractory variant of invasive carcinoma of the breast. However, because of its rarity, the most appropriate therapeutic regimens as well as prognosis still remains unclear.

## 2 CASE REPORT

A 54‐year‐old female patient presented with gradually progressive painless lump in the left breast for 3 months. She had no specific family history or any other systemic symptoms. On clinical examination, there was a firm, mobile, nontender breast lump in upper inner quadrant of the left breast measuring approximately 2.5 × 2 cm with overlying skin being normal and no axillary lymphadenopathy or swelling in the contralateral breast. Mammogram showed ill‐defined branching soft‐tissue density with scattered areas of calcification with a query of dilated duct in upper inner quadrant of the left breast. Ultrasonography revealed a cyst in upper inner quadrant measuring 3 × 2 cm containing echogenic debris along with a hypoechoic solid appearing lesion seen in continuity with its wall measuring 2.6 × 1.5 cm. Final impression suggested possibility of malignancy (BIRADS 4C) with a suspicion of intraductal lesion.

**FIGURE 1:**
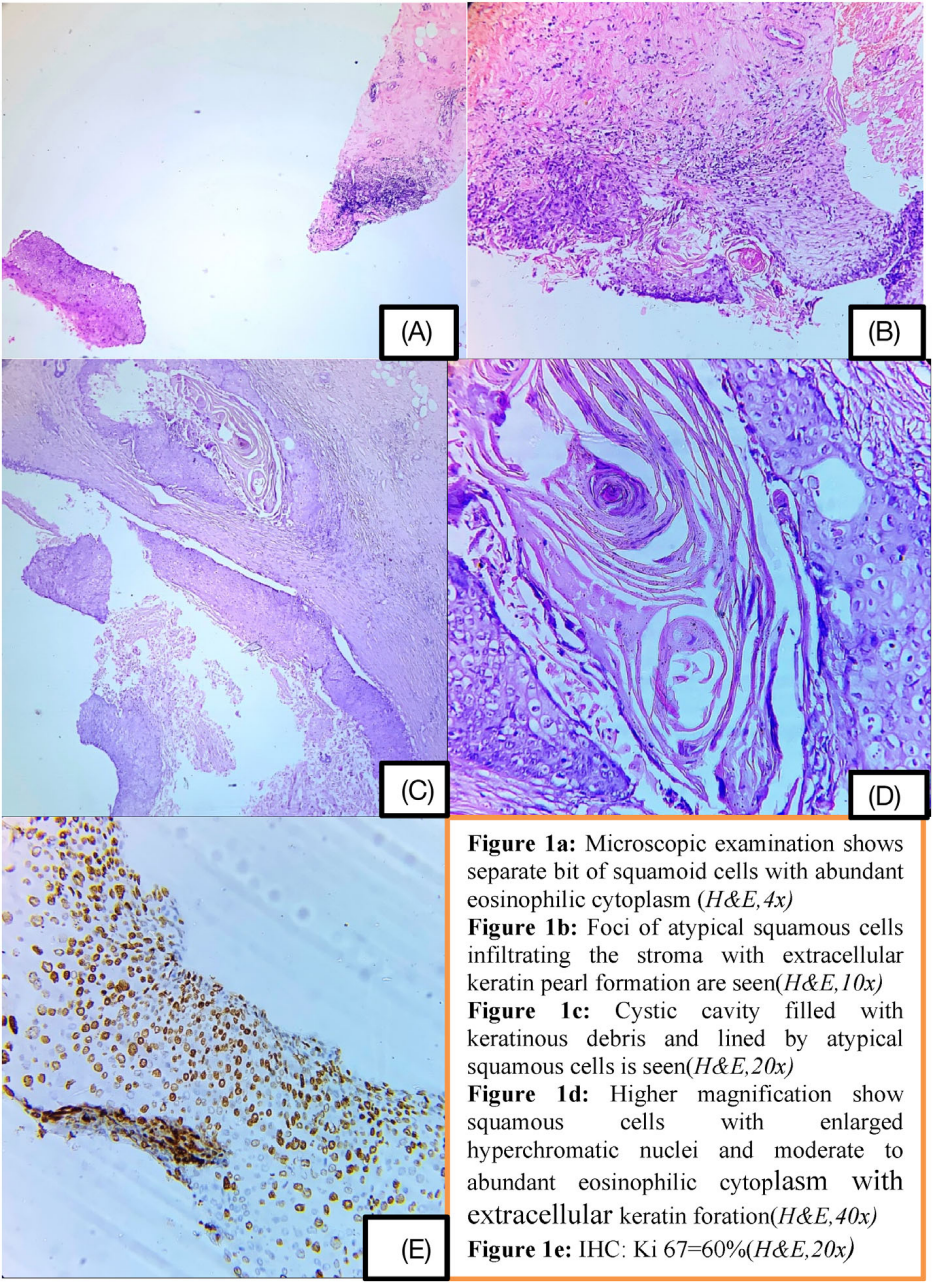
(A) Microscopic examination shows separate bit of squamoid cells with abundant eosinophilic cytoplasm (*H&E,4x*). (B) Foci of atypical squamous cells infiltrating the stroma with extracellular keratin pearl formation are seen (*H&E,10x*). (C) Cystic cavity filled with keratinous debris and lined by atypical squamous cells is seen (*H&E,20x*). (D) Higher magnification shows squamous cells with enlarged hyperchromatic nuclei and moderate to abundant eosinophilic cytoplasm with extracellular keratin foration (*H&E,40x*). (E) IHC: Ki 67 = 60% (*H&E,20x*)

Core needle biopsy was performed which showed fragmented cores composed of predominantly of fibrocollagenous tissue with benign ducts interspersed in the tissue. A separate small sheet of polygonal cells with eosinophilic cytoplasm was seen. A suspicion of malignancy was raised, and repeat biopsy from representative site was advised (Figure 1(A)). On repeat core needle biopsy, again a small focus of atypical cells of above‐mentioned morphology was seen. Some extracellular keratin could also be appreciated (Figure 1(B)). Hence, a possibility of metaplastic carcinoma with squamous differentiation was considered.

Wide local excision was performed, the cut surface of which showed a cystic lesion filled with whitish material along with presence of a grey white well‐defined firm area measuring 3 × 2.2 cm. Microscopic examination revealed a lesion filled centrally with keratinous debris and lined by atypical squamous cells, infiltrating the surrounding stroma as islands and clusters (Figure 1(C, D)). No other component was seen, and overlying skin was also not involved. Immunohistochemically, these tumor cells were negative for estrogen receptor, progesterone receptor, and her 2 neu with high‐proliferation rate (Ki 67 index 60%, Figure 1(E)). An extensive workup ruled out possibility of other primary sites or metastasis.

The patient was later planned for radical mastectomy with no neoadjuvant chemotherapy. Histopathological evaluation of MRM specimen showed presence of a microinvasive focus. All the axillary lymph nodes were negative for any tumor deposits. The postoperative phase was uneventful, and the patient is in follow‐up for last 1 year.

## 3 DISCUSSION

Primary SCC of the breast is a very rare and aggressive variant of invasive breast carcinoma and was first recorded in 1908, with very few published reports since then. Besides its uncertain prevalence, prognosis, and optimal diagnostic, as well as therapeutic management, the definition of primary SCC of the breast is also debatable. The WHO defines it as a breast carcinoma entirely composed of metaplastic squamous cells that can be keratinizing, nonkeratinizing, or tapered and not derived from overlying skin or represents metastasis.^2^ On the other hand, the Rosen's Breast Pathology defines it as a form with a squamous component comprising >90% of tumor cells.^3^ Therefore, many cases published so far may not be actually categorized as primary squamous cell carcinoma of the breast as per WHO, further causing difficulty to assess its true incidence. In our, the entire tumor component was squamous in nature.

Different theories have been proposed regarding the histogenesis of this tumor. Some authors suggest that they arise through metaplastic change in ductal carcinoma cells while others suggest their development from a chronic mastitis, breast abscess, or cyst.^4^ In our cases, histologically, a cyst was seen lined by squamous cells and filled with keratinous debris with a solid component of atypical squamous cells infiltrating the surrounding stroma along with presence of desmoplasia. Hence, a possibility of SCC arising from an epidermoid may be considered.

Literature search suggests that primary SCC of the breast usually affects postmenopausal females as rapidly growing breast lump, usually >4 cm in size with a lower rate of axillary lymph node involvement as compared to the invasive ductal carcinoma of the breast.^5,^ ^6^ Radiologically, cystic lesion is the characteristic presentation of primary SCC breast and encountered in >50% cases. Our cases also showed a cyst in upper inner quadrant measuring 3 × 2 cm containing echogenic debris along with hypoechoic solid appearing lesion seen in continuity with its wall measuring 2.6 × 1.5 cm. Hence, any cystic lesion should be evaluated adequately for presence of any associated solid component as well as irregular contour. Contradictory to previous known fact that there is lack of microcalcifications in squamous cell carcinoma breast,^7^ there was extensive microcalcifications in our case, and, hence, the patient was planned for modified radical mastectomy. Our cases was similar to majority of cases in terms of absent nodal metastasis and presence of triple negativity along with high proliferation index.

Squamous differentiation may be seen in certain malignant as well as benign breast conditions like invasive carcinomas with medullary‐like features, phyllodes tumors, breast abscess, chronic mastitis, and so forth. Apart from these conditions, possibility of metastatic SCC and SCC arising from overlying epidermis should also be ruled out. In our case, all the metastatic causes like carcinoma of lung, esophagus, skin, and so forth were ruled out both clinically and radiologically as the patient did not have any other complaint apart from painless breast lump.

The definitive management of metaplastic carcinomas is still not clear due to its rarity. Surgical management followed by chemotherapy and radiotherapy is followed as for other invasive carcinomas of the breast. Unfortunately, these tumors are relatively resistant to conventional chemotherapy. Zhang et al.^8^ and Hennessy et al.^9^ in their study also concluded that there is no role of neoadjuvant chemotherapy. Hormonal therapy is also not indicated as these tumors are triple negative. Similarly, role of adjuvant radiotherapy is also debated with no solid data. However, since the squamous cell carcinoma of other sites shows response to radiotherapy, it seems legitimate to use it in absence of specific data on management of primary SCC of the breast. Recent study has shown remarkable response to immunotherapy (anti‐PD‐L1).^10^

Basal cell subtype of breast carcinoma and primary SCC of the breast share many common features suggesting a possibility of breast SCC to be actually belonging to this group, originating from a progenitor cells in breast with a degree of maturational plasticity. However, application of gene profiling may clarify this in future.

## 4 CONCLUSION

Primary breast SCC is an aggressive, triple‐negative variant that usually affects postmenopausal elderly women. They have limited tendency for nodal metastases, usually cystic and hormone receptor negative. As our case was managed only surgically with no chemo‐radiotherapy, the only surgical excision may become main mode of treatment in the coming future. Due to rarity, their prognosis and response to current therapeutic regimen remain questionable demanding future studies to shed light.

## CONFLICT OF INTEREST

The authors have stated explicitly that there are no conflicts of interest in connection with this article.

## AUTHOR CONTRIBUTIONS

All authors had full access to the data in the study and take responsibility for the integrity of the data and the accuracy of the data analysis. *Conceptualization*, D.G., C.R., P.R.; *methodology*, D.G., C.R.; *investigation*, C.R., P.R.; *formal analysis*, D.G., C.R.; *writing—original draft*, C.R.; *writing—review and editing*, C.R., S.B., P.R.; *supervision*, C.R.; *data curation*, D.G., C.R.; *project administration*, C.R.; *validation*, C.R.

## ETHICAL STATEMENT

Consent of the patient was taken and ethical clearance was approved by the institution, King George's Medical University, Lucknow.

## Data Availability

The data will be made available by corresponding author upon reasonable request.
